# Perceptions of work stress causes and effective interventions in employees working in public, private and non-governmental organisations: a qualitative study

**DOI:** 10.1192/pb.bp.115.050823

**Published:** 2016-12

**Authors:** Kamaldeep Bhui, Sokratis Dinos, Magdalena Galant-Miecznikowska, Bertine de Jongh, Stephen Stansfeld

**Affiliations:** 1 Barts and The London School of Medicine and Dentistry; 2 BPP University, London

## Abstract

**Aims and method**

To identify causes of stress at work as well as individual, organisational and personal interventions used by employees to manage stress in public, private and non-governmental organizations (NGOs). Qualitative interviews were conducted with 51 employees from a range of organisations.

**Results**

Participants reported adverse working conditions and management practices as common causes of work stress. Stress-inducing management practices included unrealistic demands, lack of support, unfair treatment, low decision latitude, lack of appreciation, effort–reward imbalance, conflicting roles, lack of transparency and poor communication. Organisational interventions were perceived as effective if they improved management styles, and included physical exercise, taking breaks and ensuring adequate time for planning work tasks. Personal interventions used outside of work were important to prevent and remedy stress.

**Clinical implications**

Interventions should improve management practices as well as promoting personal interventions outside of the work setting.

The conceptualisation of work stress is of crucial importance when developing interventions for the workplace. Work-related stress is defined as ‘a harmful reaction that people have to undue pressures and demands placed on them at work’.^1^ As many as 440 000 people in the UK complain of work-related stress, depression or anxiety that makes them ill; nearly 9.9 million work days were lost as a consequence in 2014/2015.^1^ The most recent Health and Safety Executive (HSE) report (2015) gives a prevalence of 1380 and an incidence of 740 per 100 000 workers, and also concludes that work stress is more common in public service institutions.^1^ The estimated economic costs to the British economy as a result of stress at work are considerable, with £14.3 billion lost in 2013/2014,^2^ and the higher costs in public service amount to £1.2 billion per year.^3^

Work stress can lead to physical illness, as well as psychological distress and mental illness.^4,5,6^ The recent increase in work stress has been linked with the global and national recession,^7-9^ job insecurity and work intensity, all leading to greater workloads and more interpersonal conflicts,^3^ and can have an impact on children's mental health through disrupted parenting.^10^ Essentially, stress in the workplace may be the result of exposure to a range of work stressors and appears to arise when people attempt to manage their responsibilities, tasks or other forms of pressure related to their jobs, and encounter difficulty, strain, anxiety or worry in this attempt.^11^ Work stressors can take different forms depending on the characteristics of the workplace, and may be unique to an organisation or an industry.^12^ Theoretical models of stress consider it to be either related to adverse life events and stressful environments or the individual's physiological and psychological responses to stressors, or a ‘transactional’ interaction between the individual and environment.^13,14–17^ Although theoretical models conceptualise stress as a result of an imbalance between perceptions of external demands and internal resources, the consensus between theoretical academic models and lay representations of definitions of stress is far from clear. Definitions of stress in the research literature as well as those reported by lay people vary considerably. For example, Kinman & Jones^18^ found that there was a lack of consensus on conceptualisations of stress, and a number of different personal, social, environmental and work-related factors were used to define and interpret the meaning of stress.^11^ Brooker & Eakin^19^ suggest that concepts such as power or control in relation to gender and class are related to stress, yet models of stress do not explicitly take them into account. For example, Page *et al*^20^ found that participants perceived stress as a feminine trait associated with weakness, thus few people admit to it.

Cahil,^16^ Cooper *et al*^13^ and Marine *et al*^21^ describe categories of stress management interventions that target individuals or organisations; these can be further segmented as preventive interventions at primary, secondary or tertiary levels.^22^ Primary interventions aim to prevent the causal factors of stress, secondary interventions aim to reduce the severity or duration of symptoms, and tertiary or reactive interventions aim to provide rehabilitation and maximise functioning among those with chronic health conditions.^23^ Individual interventions may include stress awareness training and cognitive–behavioural therapy (CBT) for psychological and emotional stress. Organisational interventions affect groups of people at work and may include workplace adjustments or conflict management approaches in a specific organisation. Some interventions target both the individual and the organisation, for example policies to secure a better work–life balance and peer-support groups. Bhui *et al*'s systematic review^24^ found that interventions that target individuals show larger effects compared with organisational interventions on individual outcomes such as levels of depression and anxiety. However, individual interventions did not improve organisational outcomes such as absenteeism, which is the most important indicator of loss of organisational productivity. The evidence gaps identified in this review included studies that compared different types of organisation (e.g. public, private and non-governmental organisation (NGO)), and studies that examine whether they employ and benefit from similar interventions, given that different sectors deploy very different business processes, levels and consistency of resources and profit focus, and public service or charitable objectives. Furthermore, studies tended to be based in work settings, rather than considering all interventions applied outside of work that people found helpful.^25^

## Aims

These evidence gaps continue to exist despite the growing body of research into work stress. Our systematic synthesis of the research evidence on managing work stress showed a wide variety of organisational settings, research methods and outcome measures, such that too many questions were being asked but few answered definitively.^24^ We concluded that more empirical research was required, but that surveys were premature until there was a better understanding of:

- What work stress issues do employees and managers face on a day-to-day basis?
- What are organisations and employees already doing about work stress in the workplace and outside of work?
- What are the interventions that employees perceive to be the most effective when managing work-related stress?
- Should the approaches taken by public and private organisations and NGOs be distinct given the very different levels and consistency of resources, focus on profit, and public service or charitable objectives?

The present study used qualitative interviews to address these questions and to identify individual, organisational and personal interventions and their perceived effectiveness in managing work-related stress. Contrasts between different types of organisation were also investigated.

## Method

### Participants

The sample used in this study was purposive (e.g. type and size of organisation, vocational role) and explored participants' experiences of work stress in the course of their working day. A total of 12 organisations took part in the study, of which 6 were public organisations, 4 were private organisations and 2 were NGOs. Three organisations were based outside London, whereas the remaining 9 were based in London. Organisations were from a variety of sectors, including education, health services, insurance, graphic design and betting agencies. The purposive sampling methods aimed to ensure as broad as possible a representation of organisations, levels of the organisation and types of work within the organisation.

For the organisations that met the inclusion criteria, the senior manager was contacted by telephone and informed about the nature of the study, and agreement was reached for participation in the study. The researchers selected a number of employees in different positions along the hierarchy so there is representation from different roles: 28 of the participants were in managerial roles, and the remaining were in non-management positions. We interviewed approximately 5 participants per organisation. A total sample of 51 employees (17 men and 34 women) took part in the study; 26 participants were aged 30 years or under, 17 were between 31 and 50 years and 8 were over 51 years old.

### Procedure and topic guide

The topic guide was piloted on six employees who had experienced work stress. The content of these pilot interviews was used to refine the topic guide and to gain feedback from participants on clarity and appropriateness of the questions. Organisations were approached and invited to participate by e-mails containing information on the nature of the study and the data collection processes. Participation was voluntary; the interviews were conducted face to face and, whenever possible, at the interviewee's place of work. The interviews were semi-structured and lasted up to 45 minutes. The topic guide focused on factors that may cause stress and/or absence, personal experiences of and/or recommendations on managing stress at work and experiences of effective individual and organisational interventions to manage work-related stress (see the appendix for topic guide).

### Analysis

The interviews were transcribed verbatim, excluding any potentially identifying information. The data collected were subject to thematic analysis in order to identify and describe recurring themes.^26^ Themes and subthemes were organised using the framework approach that is commonly used in policy-relevant qualitative research.^27^ Charts were generated from the themes, the range and nature of the experiences were mapped, and patterns between and within themes were revealed. Typologies were iteratively generated to accommodate the data if existing themes were inadequate or were better grouped within a higher theme. Content analysis was also performed by counting the frequency of themes in order to identify their relative prominence in the data, and also to reveal types of interventions and contexts, and views about effectiveness. These frequencies are presented only to support the strength of the findings in these data rather than to estimate prevalence more generally. With this purpose in mind, the analysis proceeded until saturation was reached, and only themes on which saturation was reached are presented.

## Results

Data were organised by three higher themes that captured the aims of the study:

1. perceived causes of stress at work
2. individual and organisational stress management interventions and their perceived effectiveness
3. personal interventions to manage stress at work.

### Causes of stress at work

The narrative data on participants' understanding of factors that may cause stress at work suggested working conditions, management practices, nature of job, life events and financial factors (Table 1). The majority of participants (*n* = 42/51) referred to working conditions as a main source of stress. Working conditions were mainly related to factors such as workload, the physical environment (e.g. noisy offices, lack of windows, small rooms, and offices in which the temperature was either too low or too high for comfort), long working hours, heavy workloads and understaffing.

**Table 1:** Causes of stress at work

	Organisation		
Main causes of stress	Public *n* = 27	Private *n* = 13	NGO *n* = 11
Working conditions	22	10	10
Workload			
Understaffed			
Physical environment/noise			
Lack of structure in working hours			

Type/nature of job	20	11	9
Dealing with clients			
Unpredictability, unexpected difficulties			
High responsibility/demands			
Shifts/nights/travelling			

Management practices	13	10	9
Unrealistic demands, pressure, conflicting role, effort–reward imbalance			
Lack of support/appreciation, unfair treatment			
No participation in decision-making, lack of transparency			
Poor communication			

Life events	11	5	5
Work–life balance			
Family/personal issues			
Unexpected life events, sickness			

Financial factors/problems	1	2	3
Pay/benefits, lack of appreciation in salary			
Money-making goals			
Job insecurity			

NGO, non-governmental organisation.

‘We are short [staffed] so two people cannot go on holiday at the same time, so it's such an inconvenience and … we are trying to cover the days, nights and it's like wrrrr … really stressful place to be … ’ (female, 24, NGO).

Working conditions were the main cause of stress regardless of the sector people worked in; people working in the NGO or the public sector more often referred to the physical environment and workloads as relevant factors. Private sector employees more often referred to long working hours and a lack of structure to the working day.

A similar number of participants (*n* = 40) suggested that the nature of the job itself contributed to stress, with participants from private organisations and NGOs more often reporting this as a cause of stress. Participants attributed stress more specifically to a job with high unpredictability in what may be required from day to day, or a job that demands unsociable hours.

‘Shift work … I find that quite stressful because it affects my personal life because I have to work during the weekends and that's when most of my family and friends are off … ’ (female, 26, NGO).

Management practice was proposed as a cause of stress by more than half of the interviewees (*n* = 32), but was least often implicated in the public sector organisations. Participants in high managerial positions (e.g. head of unit) tended to refer to management practice as a cause of stress less often than employees in non-managerial positions. However, for all other participants (e.g. middle management positions and employees) no differences were observed. Management practice as a cause of stress related to personal style of leadership, implicating lack of warmth and support with a feeling that staff were unimportant and not respected as people; insufficient praise or confidence-building were also important omissions that caused stress. Limited opportunities for decision-making (often referred to as low decision latitude) and lack of transparency as well as unrealistic demands, poor communication and effort–reward imbalance were all implicated.

‘That's what makes you angry, because there's nobody taking my case. As soon as this phone goes to my boss and they complain about me, nobody asks me what happened. And even if I'm right, they still apologise. Why? I've done nothing wrong. It's them. They've done the wrong thing’ (male, 45, public). ‘ … the message the organisation gives to you is that you don't really matter’ (female, 40, NGO).

Life events were identified as another contributor to the level of stress experienced by employees regardless of the type of organisation they worked for (*n* = 21). Life events referred to problems with family or relationships, death and sickness, as well as trying to maintain a balance between work demands and responsibilities in the social and personal or family lives of respondents.

A small number of respondents (*n* = 6) reported financial factors as a cause of work stress; financial strain causing work stress was related to working for organisations that lacked a benefits package, or in which the salary did not reflect the amount of effort invested in work. Job insecurity as a cause of work stress reflected fears about losing income and facing further financial strain.

### Individual and organisational stress management interventions

Participants were asked about any interventions at their workplace for managing stress. Overall, participants referred less frequently to individual interventions; such interventions were also either secondary or tertiary. In particular, they were either psychological interventions such as face-to-face telephone or internet counselling, or educational interventions or training courses that taught practical skills such as organisational management and assertiveness (Table 2).

**Table 2:** Individual and organisational stress management interventions

		Organisation		
	Reported as an effective intervention	Public *n* = 27	Private *n* = 13	NGO *n* = 11
Individual: psychological interventions		5	3	3
1-2-1 therapy, counselling				
Intranet/internet counselling	✓			
Complementary therapy				
Helpline/telephone coaching				

Individual: education		1	1	0
Organisational skills courses				
Assertiveness training				

Organisational: management practices		20	8	7
Supportive, approachable, appreciative	✓			
Communicative	✓			
Frequent business/staff meetings	✓			
Supervision	✓			
2-way feedback	✓			

Organisational: organisational team culture		16	5	6
Space for discussion	✓			
Dialogue group	✓			
Pre-/post- group	✓			
Notice board	✓			

Organisational: working structure		10	0	4
Flexibility	✓			
Balanced working hours	✓			
TOIL	✓			
Fixed days off	✓			
Well-planned shifts	✓			

Organisational: education		14	4	4
Educational/training courses	✓			
Inset days	✓			

Organisational: environment		9	3	2
Open-plan office				
Relaxation room	✓			
Working room				

Organisational: health promotion		5	1	0
Gym membership	✓			
Health promotion courses				
Health promotion activities such as head massage and exercise	✓			

NGO, non-governmental organisation; TOIL, time off in lieu.

Some patterns emerged in the use of individual interventions by type of organisation: NGOs were least likely to deliver individual interventions to employees, perhaps owing to cost. In terms of effectiveness, those participants who received one-to-one counselling interventions thought these were effective, mainly because they could be accessed promptly when needed.

‘So [I] went to my [general practitioner] GP and they set it up through my local authority for face-to-face counselling, so I was seeing the occupational therapist, [had] face-to-face counselling and that happened on my day off. So [I] could do some work at home, because I was very tired I didn't have the stress of having to get into work, so just took a bit off. So [it was] kind of a package of things that just assisted me for a while’ (female, 49, public).

Organisational interventions were more often mentioned by workers in public sector employment and in contrast to individual interventions, they were mainly primary or secondary (Table 2). Most of these organisational interventions were related to management practices (*n* = 35/51). In particular, participants mentioned efforts to develop a management style that was supportive and improved communication, as well as frequent team meetings and supervision and two-way feedback.

‘[The manager is] one of those people that make you feel appreciated, even if it's a little thing she will praise you for it. A lot goes for being praised, that in itself can take away stress. If someone turns around and says what you are doing is a fantastic job you feel good’ (female, 52, public).

A supportive organisational and team culture, a collective spirit including dialogue in groups and space for discussion, and educational and training courses to improve management skills were frequently reported as effective interventions to manage stress at work (*n* = 27/51).

‘ … it's good that we all sit down together and discuss anything that may be causing a problem or tension, or anything we feel needs to be adjusted’ (female, 27, public).

Flexibility in working hours, well-planned shifts and environmental or structural interventions such as a staff room for relaxation were mentioned by almost a third of the participants as effective ways of managing work stress. Participants working in private sector organisations rarely report the existence of any interventions related to work structure (e.g. flexibility in work times), whereas in the public sector there was evidence of trying to introduce more flexibility.

‘ … time off in lieu […] seems to work quite well 'cos it's about that work–life balance’ (female, 54, public).

Almost half of participants (*n* = 22) said training and career development opportunities in the workplace were effective for managing work-related stress, as they made them feel adequately informed and valued. Appropriate training and adequate equipment and resources allowed employees to perform their roles effectively. A small number of interviewees (*n* = 3) suggested training in stress management was a useful intervention.

‘[My manager] is very good at sending people on training courses. I've just been on one which is positive interactions, which was telling you the right way of dealing with situations’ (female, 52, public).

Finally, a small number of participants (*n* = 9/51) reported that there were a number of health promotion interventions (e.g. courses, exercise) at their workplace to help them prevent work stress. Participants thought that being subsidised for gym membership or being encouraged by their organisation to exercise during their working day were very effective interventions. None of the participants working for NGOs mentioned health promotion.

‘ … we're quite actively encouraged to do lots of exercise in this trust, we get lots of emails about walking to work, or running … I think linking exercise and well-being and being healthy at work […] I think that's always good, it would be good to have that in every institution’ (female, 28, public).

### Personal interventions to manage work stress

Participants were asked general questions about their personal strategies to manage work stress. We were interested in personal interventions not provided by their employers but ones that were used and considered effective.

Table 3 shows the types of personal interventions used at work. Some interventions helped employees process stressful thoughts and think through difficult situations, akin to what CBT therapists might suggest as cognitive restructuring and tackling cognitive distortions – for example, focusing on positive rather than stressful situations, and using self-reflection to gain a better perspective.

**Table 3:** Effective personal interventions to manage stress at work (total number of respondents: 51)

Intervention	*n*
Cognitive interventions	23
Positivity/mindfulness, not focusing on problems	
Focusing on problems, self-reflection	
Replicate/learn from past performance	
Self-motivation	

Support	35
Colleague support	
Family/friend support	

Health promotion	24
Healthy eating	
Gym, exercising, sports Yoga, meditation, breathing	

Structure/organisation	26
Weekends off, breaks	
Time management, prioritising, not taking work home	
Work–personal life balance	
Keep going to work	

Interests	31
Leisure activities	
Holidays

‘Yeah, yeah or if something happens I try and think, erm, so there's another technique I learnt in the last place I worked at was five questions so I ask, the why questions five times, why this? why is it causing stress? … because of this, why is that? Why is that? And I usually get to the root cause and that usually chills me out a bit if I deal with the root cause rather than the thing causing me the stress’ (male, 30, public).

Support from colleagues and friends was the most frequently reported personal intervention for managing stress at work.

‘I actually had to say to a colleague, “I can't see the wood for the trees here, can you help me?” and the colleague was absolutely brilliant and helped me so we got through it. I got some excellent help from a colleague yeah and that was someone I worked with in the team who does the same job as me. My manager was very supportive and helpful as well’ (male, 41, private).

Keeping oneself organised and maintaining a structured schedule at work were thought to be very effective personal interventions. These included planning, reducing overtime, prioritising tasks and keeping a better balance between work and personal life.

‘One of the signals for me is if my desk starts getting a bit messy, it means I'm chasing between too many projects and that's often the time when I personally just take stock, think, right, what do I need to do, look at a priority list, clear the decks again and sort of take a bit of a step back and review. So that's how I manage it and I find that to be helpful for me’ (male, 47, public).

Almost half of the participants (*n* = 24) pointed out the importance of a healthy lifestyle when trying to manage stress at work. In particular, exercise was the most frequently reported personal intervention. Participants also acknowledge the importance of healthy eating as a means of maintaining a healthy weight and better health in general, both of which helped people to better manage stress at work.

‘Exercise is the most important thing for me for stress. So yeah, if I'm stressed, as long as I can, I'll often leave work at a decent time and go for a run and come back to work or go take my computer home and go for a swim and then do some more work. As long as I can make sure I can get some exercise in then I'm fine. It kinda works quite good 'cause I can generally take a longer lunch break and go to the gym at lunch and then, you know, work later or whatever it is. That's probably the most important flexibility for me at work is being able to have that’ (female, 32, private).

Finally, having out-of-work interests and leisure activities was reported by more than half of the participants (*n* = 31). For example, participants mentioned relaxation during lunch breaks and going on holidays as an effective personal intervention.

‘We do get inspired by going to talks and design galleries and illustration events and all that sort of thing, they're hobbies as much as they are a career but, at the same time, I think that, in a way, [this] helps to alleviate the stress levels at work because what you're doing at work is part of your hobby as well’ (male, 30, private).

## Discussion

A mixture of personal, organisational and individual interventions were reported in our study, but these are not often captured together, with emphasis often being given to workplace changes or separate public health approaches to lifestyle and physical activity.^28–30^ The majority of individual and organisational interventions reported were secondary and tertiary preventive interventions, with less emphasis on primary prevention.

High-demand and low-control situations and effort–reward imbalance related to working conditions, management style and the type of job were causing distress at work.^12,31,32^ It is also important to address management practices as one of the most significant and consistent work-related stressors. Management practice as a stressor was also more prominent in private and NGO sectors than in the public sector and in middle and low management positions than in higher management ones. Participants identified poor communication with management, unfair treatment and, above all, the feeling of not being appreciated as the biggest sources of stress for them. Furthermore, many participants highlighted working conditions, such as physical environment, unsociable working hours and under-staffing, as causes of their work stress, the harmful effects of which have been identified in previous research.^18,33^ Financial factors, mainly a lack of financial recognition by the organisation, were also reported as a cause of stress. According to Stranks,^11^ when workers experience insufficient rewards in the form of salary or amount of praise received, or are missing recognition, the feeling of devaluation might appear and can contribute to an experience of work stress.

### Interventions used by employees to manage stress at work: perceived effectiveness

Participants in the present study tended to report mainly the presence of primary and secondary organisational interventions (as opposed to individual interventions) at their workplace. With regard to individual approaches, these were mainly psychological interventions. Although there is much research that has documented the effectiveness of psychological interventions, these are usually provided at the secondary or tertiary level rather than for primary prevention.^34–36^

Organisational interventions were discussed by the participants more frequently and were also more often perceived as effective in managing stress at work than individual interventions. One of the main reasons that organisational interventions were identified as an effective way of managing stress was because they were primary interventions with the aim to modify or eliminate environmental stressors. Participants in the present study identified the organisational interventions to manage stress at work as: job redesign, change of organisational culture, encouragement of participative management, introduction of work–life balance policies, flexible working and reconstruction of the organisation as well as improvement of organisational communications.^11^ The literature on organisational interventions does not identify management practices as an intervention. The main reason may stem from the fact that management is seen as part of organisational structures rather than as potentially subject to modification to manage stress. Our findings highlighted management practices as an important workplace intervention, especially management characteristics such as open communication, supportiveness, approachability and being appreciative; these ranked the highest in terms of perceived effectiveness. Improving management practices as an intervention and introducing flexibility in working structures were much more apparent in the public sector as opposed to the private sector and the NGOs. Content analysis suggested that there may be a relationship between reported causes of stress and individual and organisational interventions. For example, stress was less often reported in the public sector because there were more management interventions than in other sectors, and these were perceived to be effective by the participants.

Most personal interventions identified by participants were related to health behaviours such as exercise, meditation and healthy eating, as well as leisure activities and social support from family and colleagues. Although personal interventions outside the workplace were not considered by the organisations, it is important to emphasise the power of such interventions and that they should be included in future intervention packages. For example, physical activity programmes have been among the few organisational interventions that show convincing effects on absenteeism in accord with our previous reviews, but physical activity could be encouraged more generally.^24,30^ By adapting organisational interventions to capitalise on and encourage personal individual interventions outside the workplace, stress management in the workplace may be less necessary or more effective where it is needed.

### Strengths and limitations

The results suggest that employees in private organisations and NGOs report more perceived causes of stress and have fewer interventions in place to help employees manage stress compared with public sector organisations, notably National Health Service (NHS) employers. We have listed potential organisational, individual and personal interventions that were used and found to be helpful. These might be tested as correlates of better workforce health and well-being and less work stress.

A limitation of the study was related to the sample characteristics. Although there were variations, especially with regard to type, size and location of the organisations involved, the sample consisted of only 12 organisations in total. A larger number of organisations would have provided us with more variety of occupations and organisation size and location, which would have given a more complete picture concerning the causes of stress and interventions between sectors. Our study is exploratory, and although these are perceived causes, the findings should not be understood in terms of epidemiological causal relationships, but rather as important ways in which workers think about and manage work stress, providing clues as to how interventions might be developed, tested and located in these work settings.

Qualitative studies offer new insights and provide the in-depth and experience-near perspectives of participants, rather than an overtheorised and superficial analysis. The findings will contribute to future in-depth work including more varied samples, as well as survey research to test for interventions that correlate with organisational measures of health and well-being. Future work should also consider how to improve management practices, as these seemed to have the most important influence on reducing work stress. More research is needed to further explore the differences between private, public and NGO sectors and different job types such as education and healthcare to examine whether they respond to the same or different intervention techniques. Finally, research needs to take into account compositional effects including the demographic characteristics of samples, and the cost effectiveness of interventions.

## Appendix

### Topic guide

General questions to start the interview

1. Tell me a few things about your job
  - How do you find your job with regard to demands, pressure, working hours, etc.?
2. Do you find your job stressful?
  - Are there any elements in your job that you find stressful?
3. What would you say are the most significant factors that can cause stress at work?
  - What are the common organisational factors that can cause stress at work?
  - What are the common individual factors that can cause stress at work?

Managing stress at work (both managing yourself and other people)

4. How do you manage yourself in terms of stress at work?
  - If you manage other people, how do you manage stress?
5. Have you had any experience of managing other people?
  - How do you manage other people's stress at work?
  - How do you deal with this?
  - What were/are the challenges?
6. What would you say are the best (individual and organisational) stress management interventions (or practices) for yourself and for other employees?
  - Can you give any examples?

Managing return to work (managing both yourself and other people)

7. Have you ever been off sick because of stress?
  - How do you manage your return to work after sickness?
8. Have you had any experience of managing someone who has been off sick because of stress?
  - How do/did you manage their return to work after sickness?
  - If no experience, how would you manage their return to work?
  - What were/are the challenges?
9. What would you say are the best (individual and organisational) interventions (or practices) for managing employee return to work after sickness leave?
  - Can you give any examples?

Policies: managing stress at work and return to work

10. Is there a policy at your place of work for managing stress at work (e.g. bullying and harassment, discrimination, working hours, compassionate leave)?
  - How has this policy been put together?
  - How effective is it in practice?
  - What are the strong and weak points of this policy, if any? Or
  - How (in what ways) would you change this policy?
11. Is there a policy at your place of work for managing return to work after sickness absence?
  - How has this policy been put together?
  - How effective is it in practice?
  - What are the strong and weak points of this policy, if any? Or
  - How (in what ways) would you change this policy?
12. What would you say are the best (individual and organisational) policies for managing employee stress?
13. What would you say are the best (individual and organisational) policies for managing employee return to work after sickness absence?
